# Bacterial superinfection in adults with COVID-19 hospitalized in two clinics in Medellín-Colombia, 2020

**DOI:** 10.1371/journal.pone.0254671

**Published:** 2021-07-13

**Authors:** Juan Carlos Cataño-Correa, Jaiberth Antonio Cardona-Arias, Jessica Paola Porras Mancilla, Marcela Tabares García

**Affiliations:** 1 Universidad de Antioquia, Medellín, Colombia; 2 Clínica CES, Medellín, Colombia; 3 Clínica Las Vega Medellín, Medellín, Colombia; Ohio State University Wexner Medical Center Department of Surgery, UNITED STATES

## Abstract

COVID-19 represents high morbidity and mortality, its complications and lethality have increased due to bacterial superinfections. We aimed to determine the prevalence of bacterial superinfection in adults with COVID-19, hospitalized in two clinics in Medellín-Colombia during 2020, and its distribution according to sociodemographic and clinical conditions. A cross sectional study was made with 399 patients diagnosed with COVID-19 by RT-PCR. We determined the prevalence of bacterial superinfection and its factors associated with crude and adjusted prevalence ratios by a generalized linear model. The prevalence of superinfection was 49.6%, with 16 agents identified, the most frequent were *Klebsiella* (*pneumoniae* and *oxytoca*) and *Staphylococcus aureus*. In the multivariate adjustment, the variables with the strongest association with bacterial superinfection were lung disease, encephalopathy, mechanical ventilation, hospital stay, and steroid treatment. A high prevalence of bacterial superinfections, a high number of agents, and multiple associated factors were found. Among these stood out comorbidities, complications, days of hospitalization, mechanical ventilation, and steroid treatment. These results are vital to identifying priority clinical groups, improving the care of simultaneous infections with COVID-19 in people with the risk factors exposed in the population studied, and identifying bacteria of public health interest.

## Introduction

In December 2019, several cases of severe pneumonia of unknown origin appeared in Wuhan-China [1], later diagnosed as coronavirus 2019 (COVID-19) disease, whose etiological agent is SARS-CoV-2 (severe acute respiratory syndrome coronavirus 2) [2,3] genus β within the family *Coronaviridae*. The World Health Organization (WHO) declared this disease as a pandemic on March 11, 2020 [4]. In Colombia, the first case was reported on March 6, 2020, in a patient from Milan-Italy [5], with an increase of cases and the highest incidence in July 2020 and January 2021 [6].

The bacterial superinfection rate and mortality rate of SARS-CoV-2 have far exceeded any other common respiratory viral syndrome [7]. The superinfection of SARS-CoV-2 with other microorganisms, especially bacteria and fungi, is a determining factor in the evolution of COVID-19, increasing the difficulties in diagnosis, treatment, and prognosis [8]. Bacterial superinfection in inpatients with COVID-19 relates to disease progression and prognosis. This situation increases the admission to intensive care units, treatment with antibiotics, and the mortality [9].

Superinfection mechanisms include respiratory system damage induced by the virus, a decrease in mucociliary clearance, and damage to the immune system [10]. In this last mechanism, the virus is capable of damaging lymphocytes, especially B cells, T cells, and NK cells, which will lead to the deterioration of the immune system during the disease. The decrease in lymphocytes and the host’s immune function is the main reason that facilitates superinfection [11]. Unfortunately, at present, it is difficult to determine the kinetics of bacterial superinfection throughout COVID-19 because there is still very little information on this interaction [12].

Different studies have analyzed the prevalence of bacterial superinfections in patients with COVID-19, finding highly heterogeneous proportions (with differences greater than 50%) attributable to clinical and epidemiological characteristics of each place as well as to the diagnostic methods and criteria used. Despite this heterogeneity in the magnitude of superinfection, previous studies describe several common risk factors, highlighting being over 60 years old are, admission to the intensive care unit, mechanical ventilation, kidney failure requiring hemodialysis, pharmacological immunosuppression (steroids or biological therapy) and prolonged hospitalization [13–15]. However, this background cannot be extrapolated to other populations, given the heterogeneity of the bacterial agents involved and the diversity in superinfection prevalence.

It is necessary to identify and to characterize the bacterial superinfections that occur during SARS-CoV-2 infection. Therefore, an analysis of hospitalized cases could improve the knowledge about the interaction of viruses and bacteria throughout the disease, and consequently, provide specific information about COVID-19 in our environment—a geographical area with climatic, social, and genetic characteristics that make it unique -. Likewise, it is important to identify the main sociodemographic and clinical factors associated with bacterial superinfection in patients with COVID-19 to improve the prioritization of potential risk groups and institutional programs for clinical and epidemiological surveillance that guide subsequent etiological studies.

The objective of this research was to determine the prevalence of bacterial superinfection in adults with COVID-19, in two clinics in Medellín-Colombia during 2020, and its distribution according to sociodemographic and clinical conditions

## Materials and methods

The study was approved by the research committee of CES and Vegas clinics (Act 022 and Act 11–2020), applying the guidelines of the Declaration of Helsinki, Resolution 8430 of the Ministry of Health of Colombia for health research, and Resolution number 1995 of 1999 that establishes the standards for the management of the clinical history. Upon admission to the hospital, the patients signed an informed consent that endorsed the use of their clinical information for research purposes.

### Type and location of the study

Cross sectional study in two tertiary care clinics in the city of Medellín, with 316 hospital beds in the general ward, 15 in the intermediate respiratory care unit (IRCU), and 45 in the ICU for COVID patients.

### Study subjects

A total of 399 adults patients admitted with a diagnosis of COVID-19 between March and August 2020, who met the following eligibility criteria: patients older than 18 years old, with a positive molecular RT-PCR SARS-CoV-2 test (*Amplification Kit* Ref 09N77-090 according to the Berlin protocol) from nasopharyngeal aspirate or swab, with an evaluation by two liaison physicians of the infectious diseases group and an infectious disease specialist, and the presence of at least one of the following risk factor that justified hospitalization: patients over 60 years of age, diabetes mellitus, cardiovascular disease (coronary artery disease or chronic heart failure), lung disease (chronic obstructive pulmonary disease, emphysema, asthma), immunosuppression (prednisone > 20 mg/day for more than 14 days, methotrexate > 0.4 mg/kg/week, or biologic therapy); or poor prognostic factors as lymphopenia <800/mm3, LDH (lactate dehydrogenase) >350 IU/l, desaturation <90%, hypoxemia with PAFI (partial arterial oxygen pressure and fraction of inspired oxygen index PaO2/FIO2) <300 mm Hg, D-dimer >1 mg/ml, elevated troponin I, EKG (electrocardiogram) altered by prolonged QTc, CRP (C-Reactive protein) >10mg/L, abnormal chest X-ray or computed tomography (basal consolidation, nodules, cavitation or pleural effusion).

### Determination of bacterial superinfection

The diagnosis of bacterial superinfection in patients admitted with COVID-19 was based on the simultaneous presence of the following three diagnostic criteria which had to be presented 48 hours or more after admission (represent as new-onset, not beginning or before of admission), to ensure that it was a superinfection (means the emerging infection during the course of illness):

1. Clinical criteria: purulent sputum, persistent fever (> 38.3 C), hemodynamic instability requiring vasopressor support, and deterioration of ventilatory parameters.
2. Paraclinical criteria: worsening of leukocytosis or leukopenia and increased procalcitonin or C-reactive protein.
3. Radiological criteria: worsening of the chest radiological pattern, or a pattern not characteristic of COVID-19 (basal consolidation, nodules, cavitation, or pleural effusion).

When these three diagnostic criteria were met, a tracheal aspirate was performed to identify the etiological agent responsible for the possible superinfection. In the cultures, the isolation of a single or predominant bacterium was considered positive, with a count equal to or greater than 106 colony-forming units/mL. Once the microbiological isolation was identified, automated sensitivity tests were performed using the VITEK-2® system (bioMerieux 8.01), following the rules of the *Clinical and Laboratory Standards Institute* (CLSI) version M100-S25, update 2020.

The microbiological isolation was not part of the diagnostic criteria; it was possible to have a diagnosis of superinfection with a negative tracheal aspirate culture since the performance of the microbiological isolation from aspirate tracheal culture ranges from about 40% to 70% [16]. In both clinics, the protocol established that if there was a suspicion of bacterial superinfection, microbiological samples were taken by tracheal aspirate, and immediately after, empirical antibiotic treatment was started according to local epidemiological data and the infectology group’s evaluation. Afterward, based on the results of the culture, the definitive antibiotic scheme was adjusted. The treatment strategy for COVID-19 in our city varied throughout the months of the study. It changed according to the findings of different published protocols, which demonstrated the uselessness of drugs such as lopinavir/ritonavir, azithromycin, hydroxychloroquine, and ivermectin, leaving only the use of dexamethasone given that in our country remdesivir or tocilizumab is not available.

Tracheal aspirates, and no bronchoalveolar lavages, were performed to protect the health personnel in charge of performing this type of procedure, instead a high sensitivity technique with low specificity [17] was chosen. This technique was also selected because in the clinics where the study was carried out, the bronchoalveolar lavages were not available at night or on weekends.

### Information gathering

Once the endorsement of the research committees of both clinics was obtained, the appearance and content of the information extraction form were validated according to the criteria of 2 medical doctors, an infectious disease specialist, an epidemiologist, and the research committee members of each clinic (10 health professionals). This form consisted of the following modules: sociodemographic data, comorbidities, complications, hospital stay, and intensive care unit (ICU) stay, mechanical ventilation, bacterial superinfections, and antibiotic treatment. One physician from each clinic was trained in the standardized way to extract patient information in an anonymized Excel file (without the name, identification number, or other data that would reveal the identification of each patient). The file was delivered to the researchers with an alphanumeric code assigned to each subject included in the study.

### Control of bias

Selection and information biases were controlled through the application of the case definition criteria by the medical team, and application of diagnostic tests for bacterial superinfection with high validity (risk of false results tend to zero) applied according to the manufacturer criteria in reference laboratories endorsed by the National Institute of Health—Ministry of Health of Colombia. Additionally, forms validated by experts were used, the process of extracting patient information from clinical histories was standardized, and double independent typing by two doctors, with logical verification, was used (no values outside the measurement ranges of the variables analyzed, or clinically incoherent values).

### Statistical analysis

The variables were described with absolute (n) and relative (%) frequencies. The general prevalence of bacterial and agent-specific superinfection was determined. The association of sociodemographic and clinical variables with bacterial superinfection was determined using Pearson Chi-square test (for nominal variables) or Chi-square test for trend (for ordinal variables). For the associated sociodemographic and clinical factors, prevalence ratios were estimated with a 95-confidence interval. Confounding variables were controlled using a multivariate generalized linear model with the logarithm transformation and binomial family (log-binomial) [18,19], in which sociodemographic and clinical factors with bivariate association with bacterial superinfection were included as independent variables. The analyzes were carried out in SPSS 25.0, taking p values less than 0.05 as significant.

### Ethical aspects

The study was approved by the Research committee of both clinics. It is classified as a no-risk study from an ethical point of view.

## Result

Of all patients, 41.9% were adults over 59 years of age and 58.9% were men; the main comorbidities were hypertension (41.6%), diabetes mellitus (23.8%), obesity (15.0%), and hypothyroidism (13%); the main complication was renal failure (20.8%). In addition, 28.8% of patients required ICU, and 26.6% invasive mechanical ventilation, with a mortality of 10.5% (Table 1).

**Table 1:** Description of the sociodemographic and clinical characteristics of the study group.

Sociodemographic and clinical	Levels	n	%
**Sociodemographic**	Age group: 60 or more years	167	41.9
	Male	235	58.9
**Comorbidities**	Hypertension	166	41.6
	Diabetes	95	23.8
	Obesity	60	15.0
	Hypothyroidism	52	13.0
	Dyslipidemia	44	11.0
	Chronic lung disease	43	10.8
	Asthma	26	6.5
	Chronic kidney disease	20	5.0
	Heart failure	23	5.8
	Immunosuppression	17	4.3
**Complications**	Renal failure without renal replacement	57	14.3
	Renal failure with renal replacement	26	6.5
	Encephalopathy	38	9.5
	Polyneuropathy in critically ill patient	33	8.3
	Myocardial dysfunction and arrhythmias	30	7.5
	Ogilvie syndrome and intestinal ischemia	9	2.3
**Other clinical conditions**	Hospital stay ≥8 days	188	47.2
	Required intensive care unit (ICU stay)	115	28.8
	Required mechanical ventilation (MV)	106	26.6
	Use of steroids	303	75.9
	Death	42	10.5

The prevalence of bacterial superinfection in patients hospitalized for COVID-19 was 49.6% (n = 198), identifying 16 different species of bacteria, with *Klebsiella* (*pneumoniae* and *oxytoca*) and *Staphylococcus aureus* being the most frequent (Table 2).

**Table 2:** The general prevalence of bacterial superinfection in patients with COVID-19 and specified by causative agent.

	n	%
General prevalence	198	49,6
*Klebsiella (pneumoniae/oxytoca)*	24	32
*Staphylococcus aureus*	18	24
*Enterobacter (cloacae / aerogenes)*	9	12
*Pseudomonas aeruginosa*	8	10,7
*Serratia marcescens*	6	8
*Haemophilus influenzae*	5	6,7
*Escherichia coli*	3	4
*Acinetobacter baumannii*	3	4
*Streptococcus pneumoniae*	2	2,7
*Streptococcus viridans*	2	2,7
*Mycobacterium tuberculosis*	1	1,3
*Burkholderia cepacia*	1	1,3
*Enterococcus faecalis*	1	1,3
*Hafnia alvei*	1	1,3

Most of the patients (62.7%) received antibiotics for one week, 60.3% as monotherapy, with Ampicillin/sulbactam being the most prescribed antibiotic (56.4%), followed by Piperacillin/tazobactam (29.9%), Meropenem (18.6%), Ciprofloxacin (16.7%) and Ceftriaxone (15.2%) (Table 3).

**Table 3:** Profile of antibiotic use in patients hospitalized for COVID-19 with bacterial superinfection.

Variable	Levels	n	%
**Days of antibiotic treatment**	1–7 days	128	62.7
	8–14 days	51	25.0
	15 or more days	25	12.3
**Number of antibiotics**	One	123	60.3
	Two	35	17.2
	Three or more	46	22.5
**Type of antibiotic treatment**	Only one	123	60.3
	Multiple (2 or more)	81	39.7
**Antibiotic used**	Ampicillin/sulbactam	115	56.4
	Piperacillin/tazobactam	61	29.9
	Meropenem	38	18.6
	Ciprofloxacin	34	16.7
	Ceftriaxone	31	15.2
	Cefepime	17	8.3
	Ceftaroline	14	6.9
	Cefazolin	12	5.9
	Linezolid	12	5.9
	Vancomycin	9	4.4
	Clindamycin	9	4.4
	Aztreonam	6	2.9
	Gentamicin	3	1.5
	Daptomycin	2	1.0
	Amikacin	2	1.0
	Anti-tuberculosis (HRZE)	2	1.0
	Moxifloxacin	1	0.5
	Trimethoprim-sulfamethoxazole	1	0.5
	Ceftazidime/avibactam	1	0.5

Among the sociodemographic and clinical factors associated with bacterial superinfections in inpatients with COVID-19, it was found that bacterial superinfection was 36% higher in subjects older than 59 years old (compared to those under the age of 60), 42% higher for people with chronic lung disease, 58% higher in immunosuppressed patients, and 38% higher in patients with acute renal failure. The clinical conditions with the strongest association with bacterial superinfection were acute renal failure requiring renal replacement therapy, hospital stay ≥8 days, ICU stay, use of steroids, and mechanical ventilation (Table 4).

**Table 4:** Factors associated with the general prevalence of bacterial superinfections in hospitalized patients for COVID-19.

Variable	Levels	n Negative	% Positive (n)	Prevalence ratio (IC95%)
Age group (in years)	<60	132	43.1(100)	
	≥60	69	58.7(98)	1.36 (1.12–1.65)**
Chronic lung disease	No	187	47.5(169)	
	Yes	14	67.4(29)	1.42 (1.12–1.80)*
Immunosuppression	No	197	48.4(185)	
	Yes	4	76.5(13)	1.58 (1.19–2.09)*
Renal failure without renal replacement therapy	No	181	47.1(161)	
	Yes	20	64.9(37)	1.38 (1.10–1.72)*
Renal failure with renal replacement therapy	No	200	46.4(173)	
	Yes	1	96.2(25)	2.07 (1.81–2.37)**
Encephalopathy	No	196	45.7(165)	
	Yes	5	86.8 (33)	1.90 (1.61–2.25)**
Polyneuropathy in critically ill patient	No	194	47.0(172)	
	Yes	7	78.8(26)	1.67 (1.36–2.06)**
Myocardial dysfunction and arrhythmias	No	192	48.0(177)	
	Yes	9	70.0(21)	1.46 (1.13–1.89)*
Hospital stay (days)	1–7	138	34.6(73)	
	≥8	63	66.5(125)	1.92 (1.56–2.37)**
ICU stay	No	175	38.4(109)	
	Yes	26	77.4(89)	2.01 (1.69–2.41)**
Mechanical ventilation	No	182	37.9(111)	
	Yes	19	82.1(87)	2.17 (1.82–2.57)**
Use of steroids	No	72	25.0(24)	
	Yes	129	57.4(174)	2.30 (1.66–3.29)**
Patient’s discharge condition	Alive	193	45.9(164)	
	Dead	8	81.0(34)	1.76 (1.46–2.12)**

*p<0.05.

**p<0.01. IC95%: 95% confidence interval.

In the multivariate adjustment, the associated variables with bacterial superinfection were comorbidity due to lung disease, complications due to encephalopathy, mechanical ventilation, hospital stay, and steroid treatment (Table 5).

**Table 5:** Adjusted prevalence ratios in a generalized linear model.

Variables of the model	Prevalence ratio (IC95%)	Wald Chi-square
Chronic lung disease (Yes/No)	2.28 (1.10–4.79)*	4.76*
Encephalopathy (Yes/No)	3.72 (1.24–11.17)*	5.51*
Mechanical ventilation (Yes/No)	3.46(1.87–3.39)**	15.65**
Hospital stay (≥8 days/1-7 days)	2.37(1.48–3.81)**	12.89**
Steroid treatment (Yes/No)	2.85(1.61–5.02)**	13.08**

*p<0.05.

**p<0.01. IC95%: 95% confidence interval.

## Discussion

Viral and bacterial superinfection, concerning seasonal influenza and *Staphylococcus aureus* cases, where the bacteria contributes significantly to worsen the prognosis in terms of morbidity and mortality, is a well described fact [20,21]. More recently, with the increase in COVID-19 cases worldwide, it has been possible to demonstrate how SARS-CoV-2 can facilitate the colonization and attachment of bacteria to the host respiratory tissue, leading to mixed infections in connection with tissue destruction caused by this virus. Similarly, bacterial superinfection can facilitate the virus systemic spread, increasing the risk of systemic infections and sepsis [22,23].

Different studies carried out in the United States and several Asian and European countries have found a highly variable prevalence of bacterial superinfection in patients diagnosed with COVID-19, ranging between 1% and 50% [24–28], which is explained by the differences in criteria and diagnostic tests used. In the present study, it was found that 49.6% had bacterial superinfection, a remarkably high percentage compared with other studies [29,30]. However, this also explains the high percentage of antibiotic prescriptions in this group of patients, an aspect that does not coincide with other studies, where the percentage of superinfection reported is much lower than the percentage of prescribed antibiotics [31,32].

In this study, a tracheal aspirate was carried out to identify the etiological agent responsible for the superinfection, identifying 16 different bacteria species, which is like the data published in other studies [33,34]. However, it is noteworthy that no fungal superinfection cases have been found, as reported in other latitudes [35,36]. Broncho alveolar lavages were not performed due to the contagion risk to the healthcare professionals who performed this procedure. After collecting the tracheal aspirate samples for culture, empirical antibiotic treatment was started according to the epidemiology of each institution and current local guidelines [37].

Among the sociodemographic and clinical factors associated with bacterial superinfection, a significant relationship was found with people older than 59 years of age (compared to those younger than 60 years old), suffering from chronic lung disease, being immunosuppressed, and having acute renal failure, which is very similar to that reported in other studies [38,39]. This relationship highlights the importance of stratifying this type of patient, considering the number and severity of risk factors related to the probability of superinfection.

The clinical conditions associated with bacterial superinfection were comorbidity due to lung disease, complications due to encephalopathy, mechanical ventilation, hospital stay, and steroid treatment, which is similar to that reported in other publications [40,41]. Based on this, a series of clinical conditions can be established to early identify the patients with the highest probability of developing superinfections throughout their hospital stay, which would reduce fatal outcomes, complications, and different sequelae of the superinfection.

With regards to antibiotic treatment, many of the treatment guidelines were extrapolated from the recommendations made by international guidelines for cases of bacterial superinfection in influenza pneumonia [17,42]. Nevertheless, this study found that 62.7% of the patients received antibiotics, mainly monotherapy with Ampicillin/sulbactam (56.4%), Piperacillin/tazobactam (29.9%), Meropenem (18.6%), Ciprofloxacin (16.7%), and Ceftriaxone (15.2%), which is like those documented in other similar studies. However, the rates of superinfection in others studies were lower than the one found in this study, but despite this, they used a large number of antibiotics directed at bacterial infections that they finally failed to demonstrate [32,43–45]. This fact should make us reflect on how the syndromic approach of this type of infections is being carried out; therefore, it is urgent to implement programs to rationalize the use of antibiotics in patients with COVID-19, to prevent the increase in the use of this type of resources, and the consequent impact on the resistance of the nosocomial microbiota [46,47].

## Conclusion

There is a high prevalence of bacterial superinfections in patients with COVID-19 who require hospitalization, mainly in those with specific comorbidities, complications, prolonged stay, mechanical ventilation, and steroid treatment, which is crucial to identify priority clinical groups, and to improve the care for these types of infections, which significantly modify the evolution of patients with COVID-19 with the risk factors exposed in the population studied.

## References

[1] Huang C, Wang Y, Li X, Ren L, Zhao J, Hu Y, et al. Clinical features of patients infected with 2019 novel coronavirus in Wuhan, China. Lancet 2020;395:497–506. doi: 10.1016/S0140-6736(20)30183-5 31986264 PMC7159299

[2] Zhu N, Zhang D, Wang W, Li X, Yang B, Song J, et al. A novel coronavirus from patients with pneumonia in China, 2019. N Engl J Med 2020;382:727–33. doi: 10.1056/NEJMoa2001017 31978945 PMC7092803

[3] Zhou P, Yang XL, Wang XG, Hu B, Zhang L, Zhang W, et al. A pneumonia outbreak associated with a new coronavirus of probable bat origin. Nature 2020;579:270–3. doi: 10.1038/s41586-020-2012-7 32015507 PMC7095418

[4] WHO. Coronavirus disease 2019 (COVID-19)—situation report—51. 2020. https://www.who.int/docs/default-source/coronaviruse/situation-reports/20200311-sitrep-51-covid-19.pdf?sfvrsn=1ba62e57_10. [Accessed March 2021].

[5] Colombia Ministry of Health and Social Protection. Colombia confirms its first case of COVID-19. https://www.minsalud.gov.co/Paginas/Colombia-confirma-su-primer-caso-de-COVID-19.aspx. [Accessed March 2021]

[6] Pan American Health Organization. COVID-19 Colombia Situation Report No. 204, January 12, 2021. https://www.paho.org/es/documentos/reporte-situacion-covid-19-colombia-no-204-12-enero-2021. [Accessed March 2021]

[7] Li R, Pei S, Chen B, Song Y, Zhang T, Yang W, et al. Substantial undocumented infection facilitates the rapid dissemination of novel coronavirus (SARS-CoV-2). Science 202;368(6490):489–93. doi: 10.1126/science.abb3221 32179701 PMC7164387

[8] Shen Z, Xiao Y, Kang L, Ma W, Shi L, Zhang L, et al. Genomic diversity of SARS-CoV-2 in Coronavirus Disease 2019 patients. Clin Infect Dis 2020;71:713–20. doi: 10.1093/cid/ciaa203 32129843 PMC7108196

[9] Martins-Filho PR, Tavares CSS, Santos VS. Factors associated with mortality in patients with COVID-19. A quantitative evidence synthesis of clinical and laboratory data. Eur J Intern Med 2020;76:97–9. doi: 10.1016/j.ejim.2020.04.043 32345526 PMC7177074

[10] Ni L, Ye F, Cheng ML, Feng Y, Deng YQ, Zhao H, et al. Detection of SARS-CoV2-specific humoral and cellular immunity in COVID-19 convalescent individuals. Immunity 2020;52:971–77.e3. doi: 10.1016/j.immuni.2020.04.023 32413330 PMC7196424

[11] Qin C, Zhou L, Hu Z, Zhang S, Yang S, Tao Y, et al. Dysregulation of immune response in patients with COVID-19 in Wuhan. China. Clin Infect Dis 2020;71:762–68. doi: 10.1093/cid/ciaa248 32161940 PMC7108125

[12] Tay MZ, Poh CM, Rénia L, MacAry PA, Ng LFP. The trinity of COVID-19: immunity, inflammation and intervention. Nat Rev Immunol 2020 Jun;20(6):363–374. doi: 10.1038/s41577-020-0311-8 32346093 PMC7187672

[13] Chen X, Liao B, Cheng L, Peng X, Xu X, Li Y, et al. The microbial coinfection in COVID-19. Appl Microbiol Biotechnol 2020 Sep;104(18):7777–85. doi: 10.1007/s00253-020-10814-6 32780290 PMC7417782

[14] Lai CC, Wang CY, Hsueh PR. Co-infections among patients with COVID-19: The need for combination therapy with non-anti-SARS-CoV-2 agents?. J Microbiol Immunol Infect 2020 Aug; 53(4):505–512. doi: 10.1016/j.jmii.2020.05.013 32482366 PMC7245213

[15] Singh V, Upadhyay P, Reddy J, Granger J. SARS-CoV-2 respiratory co-infections: Incidence of viral and bacterial co-pathogens. Int J Infect Dis 2021 Feb 25;105:617–20. doi: 10.1016/j.ijid.2021.02.087 33640570 PMC7905386

[16] Ewig S, Torres A, Angeles Marcos M, Angrill J, Raño A, de Roux A, et al. Factors associated with unknown aetiology in patients with community-acquired pneumonia. Eur Respir J 2002;20:1254–62. doi: 10.1183/09031936.02.01942001 12449182

[17] Metlay JP, Waterer GW, Long AC, Anzueto A, Brozek J, Crothers K, et al. Diagnosis and treatment of adults with Community-acquired Pneumonia. An official clinical practice guideline of the American Thoracic Society and Infectious Diseases Society of America. Am J Respir Crit Care Med 2019 Oct 1;200(7):e45–e67. doi: 10.1164/rccm.201908-1581ST 31573350 PMC6812437

[18] Skov T, Deddens J, Petersen MR, Endahl L. Prevalence proportion ratios: estimation and hypothesis testing. Int J Epidemiol 1998;27:91–5. doi: 10.1093/ije/27.1.91 9563700

[19] Thompson ML, Myers JE, Kriebel D. Prevalence odds ratio or prevalence ratio in the analysis of cross-sectional data: What is to be done?. Occup Environ Med 1998;55:272–7. doi: 10.1136/oem.55.4.272 9624282 PMC1757577

[20] van der Sluijs KF, van der Poll T, Lutter R, Juffermans NP, Schultz MJ. Bench-to-bedside review: bacterial pneumonia with influenza-pathogenesis and clinical implications. Crit Care 2010;14(2):219. doi: 10.1186/cc8893 20459593 PMC2887122

[21] Bosch AATM, Biesbroek G, Trzcinski K, Sanders EAM, Bogaert D. Viral and Bacterial Interactions in the Upper Respiratory Tract. PLoS Pathog 2013;9(1):e1003057. doi: 10.1371/journal.ppat.1003057 23326226 PMC3542149

[22] Tay MZ, Poh CM, Rénia L, MacAry PA. The trinity of COVID-19: immunity, inflammation and intervention. Nat Rev Immunol 2020 Apr 28: 1–12. doi: 10.1038/s41577-020-0311-8 32346093 PMC7187672

[23] Mirzaei R, Goodarzi P, Asadi M, Soltani A, Aljanabi HAA, Jeda AS, et al. Bacterial co‐infections with SARS‐CoV‐2 IUBMB Life 2020 Aug 8: doi: 10.1002/iub.2356 32770825 PMC7436231

[24] Zhou F, Yu T, Du R, Fan G, Liu Y, Liu Z, et al. Clinical course and risk factors for mortality of adult inpatients with COVID-19 in Wuhan, China: a retrospective cohort study. Lancet 2020 28 March-3 April;395(10229):1054–62. doi: 10.1016/S0140-6736(20)30566-3 32171076 PMC7270627

[25] Arentz M, Yim E, Klaff L, Lokhandwala S, Riedo FX, Chong M, et al. Characteristics and outcomes of 21 critically ill patients with COVID-19 in Washington State. JAMA. 2020 Apr 28;323(16):1612–14. doi: 10.1001/jama.2020.4326 32191259 PMC7082763

[26] Chen N, Zhou M, Dong X, Qu J, Gong F, Han Y, et al. Epidemiological and clinical characteristics of 99 cases of 2019 novel coronavirus pneumonia in Wuhan, China: a descriptive study. Lancet 2020;15–21 February;395(10223):507–13. doi: 10.1016/S0140-6736(20)30211-7 32007143 PMC7135076

[27] Young BE, Ong SWX, Kalimuddin S, Low JG, Tan SY, Loh J, et al. Epidemiologic features and clinical course of patients infected with SARS-CoV-2 in Singapore. JAMA 2020 Apr 21;323(15):1488–94. doi: 10.1001/jama.2020.3204 32125362 PMC7054855

[28] Grasselli G, Zangrillo A, Zanella A, Antonelli M, Cabrini L, Castelli A, et al. Baseline Characteristics and Outcomes of 1591 Patients Infected With SARS-CoV-2 Admitted to ICUs of the Lombardy Region, Italy. JAMA 2020 Apr 28;323(16):1574–81. doi: 10.1001/jama.2020.5394 32250385 PMC7136855

[29] Langford BJ, So M, Raybardhan S, Leung V, Westwood D, MacFadden DR, et al. Bacterial co-infection and secondary infection in patients with COVID-19: a living rapid review and meta-analysis. Clin Microbiol Infect 2020 Dec;26(12):1622–29. doi: 10.1016/j.cmi.2020.07.016 32711058 PMC7832079

[30] Lansbury L, Lim B, Baskaran V, Lim WS. Co-infections in people with COVID-19: a systematic review and meta-analysis. J Infect 2020 Aug;81(2):266–75. doi: 10.1016/j.jinf.2020.05.046 32473235 PMC7255350

[31] Townsend L, Hughes G, Kerr C, Kelly M, O’Connor R, Sweeney E, et al. Bacterial pneumonia coinfection and antimicrobial therapy duration in SARS-CoV-2 (COVID-19) infection. JAC Antimicrob Resist 2020 Sep;2(3):dlaa071. doi: 10.1093/jacamr/dlaa071 32864608 PMC7446659

[32] Goncalves Mendes Neto A, Lo KB, Wattoo A, Salacup G, Pelayo J, DeJoy R 3rd, et al. Bacterial infections and patterns of antibiotic use in patients with COVID-19. J Med Virol 2021 Mar;93(3):1489–95. doi: 10.1002/jmv.26441 32808695 PMC7461450

[33] Nori P, Cowman K, Chen V, Bartash R, Szymczak W, Madaline T, et al. Bacterial and fungal coinfections in COVID-19 patients hospitalized during the New York City pandemic surge. Infect Control Hosp Epidemiol 2021 Jan;42(1):84–8. doi: 10.1017/ice.2020.368 32703320 PMC7417979

[34] Zhu X, Ge Y, Wu T, Zhao K, Chen Y, Wu B, et al. Co-infection with respiratory pathogens among COVID-2019 cases. Virus Res 2020 Aug;285:198005. doi: 10.1016/j.virusres.2020.198005 32408156 PMC7213959

[35] Hughes S, Troise O, Donaldson H, Mughal N, Moore LSP.Bacterial and fungal coinfection among hospitalized patients with COVID-19: a retrospective cohort study in a UK secondary-care setting. Clin Microbiol Infect 2020 Oct;26(10):1395–1399. doi: 10.1016/j.cmi.2020.06.025 32603803 PMC7320692

[36] Rawson TM, Moore LSP, Zhu N, Ranganathan N, Skolimowska K, Gilchrist M, et al. Bacterial and fungal co-infection in individuals with coronavirus: a rapid review to support COVID-19 antimicrobial prescribing. Clin Infect Dis 2020 Dec 3;71(9):2459–68. doi: 10.1093/cid/ciaa530 32358954 PMC7197596

[37] Colombian Association of Pulmonology and Chest Surgery (ACNCT), Colombian Association of Critical Medicine and Intensive Care (AMCI), Colombian Association of Internal Medicine (ACMI), Colombian Association of Infectology (ACIN). Recommendations for diagnosis, treatment and prevention of community-acquired pneumonia in immunocompetent adults. Infectio 2013;17(Supl 1):1–38. doi: 10.1016/S0123-9392(13)70019-5

[38] Richardson S, Hirsch JS, Narasimhan M, Crawford JM, McGinn T, Davidson KW, et al. Presenting Characteristics, Comorbidities, and Outcomes Among 5700 Patients Hospitalized With COVID-19 in the New York City Area. JAMA 2020 May 26;323(20):2052–9. doi: 10.1001/jama.2020.6775 32320003 PMC7177629

[39] Lv Z, Cheng S, Le J, Huang J, Feng L, Zhang B, et al. Clinical characteristics and co-infections of 354 hospitalized patients with COVID-19 in Wuhan, China: a retrospective cohort study. Microbes Infect 2020 May-Jun;22(4–5):195–9. doi: 10.1016/j.micinf.2020.05.007 32425649 PMC7233257

[40] Ripa M, Galli L, Poli A, Oltolini C, Spagnuolo V, Mastrangelo A, et al. Secondary infections in patients hospitalized with COVID-19: incidence and predictive factors. Clin Microbiol Infect 2021 Mar;27(3):451–7. doi: 10.1016/j.cmi.2020.10.021 33223114 PMC7584496

[41] Buckner FS, McCulloch DJ, Atluri V, Blain M, McGuffin SA, Nalla AK, et al. Clinical Features and Outcomes of 105 Hospitalized Patients With COVID-19 in Seattle, Washington. Clin Infect Dis 2020 Nov 19;71(16):2167–73. doi: 10.1093/cid/ciaa632 32444880 PMC7314181

[42] Uyeki TM, Bernstein HH, Bradley JS, Englund JA, File TM, Fry AM, et al. Clinical practice guidelines by the Infectious Diseases Society of America: 2018 update on diagnosis, treatment, chemoprophylaxis, and institutional outbreak management of seasonal influenza. Clin Infect Dis 2019 Mar 15;68(6):895–902. doi: 10.1093/cid/ciy874 30834445 PMC6769232

[43] Liu C, Wen Y, Wan W, Lei J, Jiang X. Clinical characteristics and antibiotics treatment in suspected bacterial infection patients with COVID-19. Int Immunopharmacol 2021 Jan;90:107157. doi: 10.1016/j.intimp.2020.107157 33187911 PMC7608018

[44] Karami Z, Knoop BT, Dofferhoff ASM, Blaauw MJT, Janssen NA, van Apeldoorn M, et al. Few bacterial co-infections but frequent empiric antibiotic use in the early phase of hospitalized patients with COVID-19: results from a multicentre retrospective cohort study in The Netherlands. Infect Dis (Lond) 2021 Feb;53(2):102–110. doi: 10.1080/23744235.2020.1839672 33103530

[45] Seaton RA, Gibbons CL, Cooper L, Malcolm W, McKinney R, Dundas S, et al. Survey of antibiotic and antifungal prescribing in patients with suspected and confirmed COVID-19 in Scottish hospitals. J Infect 2020 Dec;81(6):952–60. doi: 10.1016/j.jinf.2020.09.024 32987097 PMC7518971

[46] Bengoechea JA, Bamford CG. SARS-CoV-2, bacterial co-infections, and AMR: the deadly trio in COVID-19? EMBO Mol Med 2020 Jul 7;12(7):e12560. doi: 10.15252/emmm.202012560 32453917 PMC7283846

[47] Sieswerda E, de Boer MGJ, Bonten MMJ, Boersma WG, Jonkers RE, Aleva RM, et al. Recommendations for antibacterial therapy in adults with COVID-19—an evidence based guideline. Clin Microbiol Infect 2021 Jan;27(1):61–6. doi: 10.1016/j.cmi.2020.09.041 33010444 PMC7527308

